# Piloting a Developmental Screening Tool Adapted for East African Children

**DOI:** 10.3390/children5080101

**Published:** 2018-07-26

**Authors:** Mollika A. Sajady, Christopher J. Mehus, Emily C. Moody, Ericka G. Jaramillo, Ezekiel Mupere, Andrew J. Barnes, Sarah E. Cusick

**Affiliations:** 1 Developmental-Behavioral Pediatrics, Division of General Pediatrics and Adolescent Health, Department of Pediatrics, University of Minnesota Medical School, Minneapolis, MN 55455, USA; drbarnes@umn.edu; 2 Division of General Pediatrics and Adolescent Health, Department of Pediatrics, University of Minnesota Medical School, Minneapolis, MN 55455, USA; cjmehus@umn.edu; 3 Environmental Medicine, Icahn School of Medicine at Mount Sinai, New York, NY 10029, USA; emily.moody@mssm.edu; 4 Icahn School of Medicine at Mount Sinai, New York, NY 10029, USA; ericka.jaramillo@icahn.mssm.edu; 5 Paediatrics and Child Health, Makerere University, Kampala, Uganda; mupez@yahoo.com; 6 Department of Pediatrics, Division of Global Pediatrics, University of Minnesota Medical School, Minneapolis, MN 55455, USA; scusick@umn.edu

**Keywords:** development, milestones, screening, poverty, stunting, lead exposure, developmental risk, child health, global health, pediatrics

## Abstract

There is a need for developmental screening that is easily administered in resource-poor settings. We hypothesized that known risk factors would predict failed developmental screening on an adapted screening tool in East African children living in poverty. The sample included 100 healthy Ugandan children aged 6–59 months. We adapted a parent-reported developmental screener based on the Child Development Review chart. The primary outcome was failure to meet age-appropriate milestones for any developmental domain. Venous blood was analyzed for lead, and caregivers completed a demographics questionnaire. We used multivariate logistic regression models to determine if elevated blood lead and stunting predicted failure on the screener, controlling for maternal education level, age in months past the lower bound of the child’s developmental age group, and absence of home electricity. In the sample, 14% (*n* = 14) of children failed one or more milestones on the screener. Lead levels or stunting did not predict failing the screener after controlling for covariates. Though this tool was feasibly administered, it did not demonstrate preliminary construct validity and is not yet recommended for screening in high-risk populations. Future research should include a larger sample size and cognitive interviews to ensure it is contextually relevant.

## 1. Introduction

Worldwide it is estimated that 200 million children under 5 years of age do not reach their developmental potential annually [1]. Nearly 80% of children with disabilities live in low- and middle-income countries [2,3]. Known risk factors that are associated with poor developmental outcomes include nutritional stunting, inadequate cognitive stimulation, and lead exposure [4]. Furthermore, previous studies in Africa reveal that delayed achievement of developmental milestones can be predicted by identifying stunting in high risk populations [5,6,7].

Children who experience poor nutrition early in life are more likely to have growth stunting [8,9]. In turn, stunting in children is also known to cause persistent cognitive deficits [10]. Also, the burden of nutritional deficiencies falls heavily on low and middle-income regions; as approximately 90% of individuals with undernutrition live in developing countries [11]. In Uganda, it is estimated that undernutrition contributes to 40% of the child mortality rate for those 5 years of age and younger [12].

The association of environmental lead exposure and poor neurocognitive outcomes is also well established [13]. Even chronic, low-level lead exposure can lead to lower IQ scores, deficits in attention, and slowed growth in children [14]. The highest risks of lead exposure in Africa include deteriorated house paint, leaded gasoline, mining operations, polluted waters, contaminated foods, and cosmetics [15]. Young children are particularly vulnerable to the neurotoxic effects of lead due to the higher frequency of hand-to-mouth behaviors and rapid changes in brain development [16]. One study in Kampala, Uganda found that approximately 20 percent of 4–8 year-old school children have blood lead levels above 7.15 μg/dL [17].

Child development screening is limited or non-existent in many low- and middle-income countries [18]. In these countries, there has been a marked decline in child mortality in recent years, but the prevalence of children living with neurodevelopmental disabilities continues to rise [18]. Children in East Africa do not routinely receive pediatric well care visits and most children with developmental disabilities are not identified until school age [19,20]. Some comprehensive developmental measures, such as the Mullen or the Bayley Scales of Development, have previously been utilized in high-risk global populations, but these can be time-intensive and require additional training to administer [21,22].

It is uncertain whether developmental screening tools standardized in Western industrialized nations are valid across cultures. Western developmental milestone tools adapted for use in African settings tend to be more reliable for gross motor items, compared to social and language development [23]. There are emerging developmental assessment tools that have been designed for use in low- and middle-income countries, however, many of these are tools are limited to children under 3 years of age. For example, the Caregiver-Reported Early Developmental Instruments (CREDI) measure of child development is a brief tool of early developmental progress in children of 0–35 months, though for study purposes, a longer version is recommended [24]. The Kilifi Developmental Inventory has been previously validated to assess psychomotor functioning of children in Kenya for ages 6–35 months [25]. The Guide for Monitoring Child Development is a clinician-caregiver interview designed in Turkey, which has been used to detect early developmental difficulties for children less than two years [26]. The Developmental Milestones Checklist is a 66-item caregiver interview designed in Kenya to assess motor, language, and social development of children aged 3–24 months [27]. Additionally, the Malawi Developmental Assessment Tool is a culturally relevant developmental assessment tool for use in rural Africa for children aged 0–6 years, however, it can be lengthy to administer with 136 items across four developmental domains and requires the use of specific objects or props for administration [28].

To date, no studies have been conducted on adapted developmental screening instruments that are easily administered in clinical or community settings and are appropriate for high-risk populations aged 6–59 months in East Africa. To begin to answer these questions, we implemented an adapted developmental screening tool to determine if known neurodevelopmental risk factors, specifically lead exposure and undernutriton as assessed by nutritional stunting, are associated with delayed developmental milestones. We piloted the screening tool as part of a larger survey on environmental heavy metal exposure among children living in the Katanga urban settlement [29]. We hypothesized that elevated blood lead levels and growth stunting would be positively associated with delayed developmental outcomes for chronological age.

## 2. Materials and Methods

### 2.1. Study Design and Participants

As previously described [29], we recently conducted a cross-sectional study of blood levels of heavy metals in 100 children aged 6–59 months living in the Katanga urban settlement of Kampala. Briefly, we mapped the Katanga area with a Global Positioning System (GPS). Every other home was approached by the study staff until a convenience sample of 100 children aged 6–59 months was reached. Inclusion criteria were: age 6–59 months, permanent resident of the Katanga settlement, and child and caretaker willing to come to Mulago Hospital on the same day for a physical exam, blood draw, and environmental questionnaire. Exclusion criteria included any child found to need urgent medical attention and the caretaker not being able to complete questionnaires in English or the local language, Luganda. Any child needing urgent medical attention was excluded from the study and immediately transported to Mulago Hospital for further care.

With the assistance of study staff, each child and his or her caregiver were guided to Mulago Hospital where informed consent was obtained, a venous blood sample was collected, and a medical history form was completed that included the adapted developmental screening tool.

Blood lead was measured in whole blood samples at the Senator Frank R. Lautenberg Environmental Health Sciences Trace Metal Laboratory at the Mount Sinai School of Medicine by LC-tandem mass spectrometry.

### 2.2. Developmental Screening Tool

To assess the developmental status of the children in our sample, we created a brief chart for age-related developmental milestones that could be administered in a caregiver-interview format, which has previously been found to be the most appropriate method of questionnaire administration for populations with lower literacy levels [30]. The purpose of piloting a brief, one-page developmental tool in lieu of other validated measures was to potentially provide a screening measure that could be applied to community level settings for East African children of 6–59 months without the need for extensive administrative training or materials. The chart was adapted from the Child Development Review chart by Harry Ireton, the Center for Disease Control milestones, and the Handicap International Developmental Chart [31,32,33]. The screening tool was created by selecting one main skill to assess for each developmental domain per age group that was most common across all of the resources. These milestone questions were modeled in the format of the Child Development Review Chart. To ensure that the language on the screener was culturally appropriate for the population, two Ugandan study coordinators reviewed the tool. The adapted developmental screening tool included eight age groups (6 months, 9 months, 12 months, 18 months, 24 months, 36 months, 48 months, and 60 months). The final tool comprised six different developmental domains: gross motor, fine motor, language, social skills, vision and hearing, and self-care. For each age group and developmental milestone, the chart listed yes or no options for the parents to answer regarding their child’s current developmental capabilities (Table 1). The study coordinators read each column of developmental milestones out loud to the caregivers until the caregiver reported that the child was not able to complete the milestone for a particular age. The child’s developmental age for each column was recorded by using the highest age for which the parent reported, “yes”, in each category. The chronological age of the child was then compared to the developmental age attained for each domain to determine the presence or absence of delay. This measure was dichotomized (pass = 0, fail = 1).

### 2.3. Ethical Considerations

Caregivers of all participants provided written informed consent. The study protocol was approved by the Institutional Review Boards of the University of Minnesota (IRB #1511M80562, approval date 2 February 2016), the Research Ethics Committee of the Makerere University School of Biomedical Sciences, and the Uganda National Council of Science and Technology (IRB #SBS 325, approval date 7 March 2016).

### 2.4. Statistical Analysis

We first conducted descriptive and bivariate analyses. We then constructed multivariate logistic regression models to determine if known factors associated with child development—specifically, blood lead levels and growth stunting—predicted failure on the developmental screening tool. Venous blood lead was measured as a continuous variable. For reference, elevated blood lead was defined as >5 μg/dL. Growth stunting was defined as height-for-age Z score of less than 2 standard deviations of the reference mean (Epi Info version 3.5.3; Centers for Disease Control and Prevention) [34]. Failure on the developmental screening tool was defined as obtaining a developmental milestone for any domain at a younger age group compared to chronological age. Separate models were constructed for blood lead and growth stunting. In each, we controlled for maternal education level, age in months past the lower bound of the child’s chronological age group on the screener, and absence of home electricity as a proxy for economic status. Three children with missing data on predictor variables were excluded from the logistic regression analysis. Each of these children passed the developmental screening tool.

## 3. Results

In this sample, 53 out of 100 children were male and the average age was slightly more than two years (Table 2). The majority of the mothers had completed primary school or lower, and slightly less than half of children had no electricity in the home. A majority of the children had elevated blood lead levels (n = 63) and slightly less than a quarter had growth stunting. Fourteen out of the 100 children failed one or more age-equivalent developmental milestones on the screening tool. Of those who failed the screening tool, seven children failed one domain with average lead level of 7.61 µg/dL (SD = 2.69) and height-for-age Z score of −1.35 (SD = 0.60), two children failed two domains with average lead level of 8.23 µg/dL (SD = 0.30) and height-for-age Z score of −1.77 (SD = 1.78), one child failed five domains with a lead level of 4.55 µg/dL and height-for-age Z score of 1.07, and four children failed all six domains with average lead level of 6.90 µg/dL (SD = 2.30) and height-for-age Z score of −1.46 (SD = 1.77). Bivariate correlations are shown in Table 3. Notably, older children were more likely to have elevated blood lead levels, in addition to higher number of months past the lower bound of the child’s age category. Failure on the screening tool was negatively correlated with lead levels and positively correlated with stunting; however, these correlations failed to reach significance and all of the bivariate correlations were close to zero. Neither elevated lead levels nor stunting predicted failing the screener after controlling for covariates (Table 4).

## 4. Discussion

We applied an adapted and easily administered screening tool designed to identify developmental milestones of children living in the Katanga urban settlement in Kampala, Uganda. This screening tool identified 14% of children in the study as having potential developmental delays. Comparatively, in the United States, approximately 15% of children between the ages of 3 years and 17 years have a developmental disability [35]. Contrary to our hypotheses, children’s developmental outcomes were not predicted by their blood lead levels or by height-for-age Z-score less than 2 standard deviations below the reference mean, which are two known correlates of developmental delays. However, there may be other correlates associated with delayed developmental milestones on this screening tool that have yet to be analyzed and the results may vary with a larger sample size. Another implication of these findings is that this screening tool may not have sufficient sensitivity and specificity to accurately detect true developmental delays in this population. Possible explanations for this include cultural and linguistic discrepancies between the screener and its target population.

We adapted the tool from widely used developmental screeners in the United States, including the Child Development Inventory (CDI). Similar tools have previously been adapted for use in East Africa as a low cost option for developmental screening, however these are typically time-consuming and require significant cultural modifications related to wording [36]. Our results also show that there are potential limitations of using developmental tools from wealthy Westernized nations in low- and middle-income countries. The populations of low- and middle-income countries may have lower parental education and health literacy, as well as differences in family structure. For instance, in East Africa, where extended families often live in shared homes with other families, there tends to be more emphasis on social and emotional security than structured cognitive activities, and young children often spend more time interacting with older children than with adults [36]. Given these unique social contexts, developmental screening tools created for Western cultures, may not capture normative development patterns specific to East Africa.

A limitation of this study is the small convenience sample of caregiver volunteers and the subsequent limited generalizability to other populations in East Africa. Other significant limitations include: reliance on caregiver-reported milestones and no requirement for primary caregiver participation in the study; the fact that we did not collect a concurrent gold-standard measure of child development with which to determine criterion validity of the screener; the lack of cognitive interviews with study participants to ensure the screeners’ contextual and cultural relevance; and the lack of inter-rater reliability for the study coordinators who administered the screener. Though the two local study coordinators who administered the items on the screening tool by verbal interview were fluent in both Luganda and English, possible communication issues or varying levels of comprehension should be considered, particularly given the wide range in educational backgrounds of the caregivers. Future studies of developmental screening in this population should correlate findings of the developmental screening tool with additional measures of neurodevelopmental risk factors, such as maternal and child health concerns. It was not possible at the time of this study inception to complete a prospective design with the primary outcome measure of developmental skills assessment given that this study was completed as part of a secondary data analysis to a larger cross-sectional study on blood levels of heavy metals in children. Future research should be completed on this screening tool with a prospective study design, controlling for moderators, such as stunting and elevated lead levels, along with a comparison gold-standard developmental assessment to better determine its validity prior to widespread use in community settings.

To meet the need for accurate, brief, culturally appropriate methods to universally screen children’s development in high-risk global populations, more culturally flexible, validated screening tools are needed. These efforts should be paired with a focus on promoting therapeutic interventions for East African children. Addressing undernutrition, maximizing cognitive stimulation, and establishing community-based therapy efforts are effective, low-cost strategies that are likely to enhance child development [26]. These efforts could be initiated by maximizing existing resources and networks, such as educating parents and community health workers on how to promote improved developmental outcomes for all children. 

## Figures and Tables

**Table 1:** Developmental screening tool.

Age	Gross Motor		Fine Motor		Language		Social		Vision/Hearing		Self-Care	
6 months	Begin to sit w/o support and roll?		Brings objects to mouth?		Repeats simple sounds (ah, ga)?		Responds to sounds/gestures?		Enjoys bright or moving objects?		Beginning to eat semi-solid foods?	
	**Y**	**N**	**Y**	**N**	**Y**	**N**	**Y**	**N**	**Y**	**N**	**Y**	**N**
9 months	Crawling?		Start to pick things up w/thumb & index fingers?		Understands no?		Afraid of strangers?		Looks for hidden or fallen objects?		Transfers objects from one hand to the other?	
	**Y**	**N**	**Y**	**N**	**Y**	**N**	**Y**	**N**	**Y**	**N**	**Y**	**N**
1 year	Stand and walk with some assistance?		Can take things out of a container?		Repeat single words (mama, dada)?		Copies simple actions (waves bye)?		Enjoys music?		Drinks on own (cup)?	
	**Y**	**N**	**Y**	**N**	**Y**	**N**	**Y**	**N**	**Y**	**N**	**Y**	**N**
18 months	Can walk alone?		Feeds self with spoon?		Says several single words & can say no?		Points to show others something interesting?		Understands 1 step commands (sit down)?		Can undress with help?	
	**Y**	**N**	**Y**	**N**	**Y**	**N**	**Y**	**N**	**Y**	**N**	**Y**	**N**
2 years	Climbs on objects and starts to run?		Stack objects?		Uses 2–3 word sentences?		Starts to play beside other children?		Copies actions of adults and other children?		Uses stairs holding on to the wall?	
	**Y**	**N**	**Y**	**N**	**Y**	**N**	**Y**	**N**	**Y**	**N**	**Y**	**N**
3 years	Runs and climbs easily?		Draws circle?		Asks/answers simple questions?		Separates from parents easily?		Sort objects and names most familiar things?		Says first name, age, and sex?	
	**Y**	**N**	**Y**	**N**	**Y**	**N**	**Y**	**N**	**Y**	**N**	**Y**	**N**
4 years	Stands on one foot for up to 2 s?		Can pour liquid and mash food?		Sing songs and/or can state first and last name?		Would rather play w/others than alone?		Follows 2–3 directions at a time?		Names some colors and numbers?	
	**Y**	**N**	**Y**	**N**	**Y**	**N**	**Y**	**N**	**Y**	**N**	**Y**	**N**
5 years	Stands on one foot for 10 s or hops?		Copies a triangle and other shapes?		Can tell stories and describe things?		Plays group games & wants to be like friends?		Listens to explanations?		Can use the toilet alone?	
	**Y**	**N**	**Y**	**N**	**Y**	**N**	**Y**	**N**	**Y**	**N**	**Y**	**N**

Sources: Ireton Child Development Chart [31], CDC Milestones [32], Handicap International Developmental Chart [33]; Y= Yes, N= No.

**Table 2:** Sample characteristics.

Category	Total
n	100
Male, n (%)	53 (53.0)
Failed one or more domain on screener, n (%)	14 (14.0)
Without electricity, n ^1^ (%)	42 (42.4)
Mean age, mos ^2^	28.51 (15.1)
Mother’s education level, n ^3^ (%)	
None	9 (9.2)
Lower primary school	14 (14.3)
Upper primary school	42 (42.9)
Lower secondary school	26 (26.5)
Upper secondary school	3 (3.1)
Tertiary school (college and above)	4 (4.1)
WHO height-for-age Z-score < −2, n ^4^ (%)	23 (23.5)
Blood lead > 5 μg/dL, n (%)	63 (63.0)
Blood lead, µg/dL ^2^	6.1 (2.6)

**^1^** Without electricity n = 99, ^2^ Mean (SD), ^3^ Mother’s education level n = 98, ^4^ WHO height-for-age Z-score n = 98.

**Table 3:** Correlations.

		Age	Without Electricity	Mother’s Education Level	Months Past Lower Bound of Age Category	Blood Lead	WHO Height-for-Age Z-Score < −2	Failed One or More Developmental Domain
Age	Pearson Correlation	1						
	Sig. (2-tailed)							
Without electricity	Pearson Correlation	−0.191	1					
	Sig. (2-tailed)	0.059						
Mother’s education level	Pearson Correlation	−0.155	0.151	1				
	Sig. (2-tailed)	0.127	0.141					
Months past lower bound of age category	Pearson Correlation	0.582	−0.123	−0.056	1			
	Sig. (2-tailed)	0.000 **	0.225	0.582				
Blood lead	Pearson Correlation	0.308	−0.025	0.088	0.086	1		
	Sig. (2-tailed)	0.002 **	0.807	0.386	0.396			
WHO height-for-age Z-score < −2	Pearson Correlation	−0.004	−0.040	−0.101	−0.045	−0.099	1	
	Sig. (2-tailed)	0.966	0.696	0.327	0.663	0.331		
Failed one or more developmental domain	Pearson Correlation	−0.099	−0.106	0.071	0.137	−0.176	0.156	1
	Sig. (2-tailed)	0.328	0.295	0.489	0.175	0.80	0.124	

** *p* < 0.05.

**Table 4:** Logistic regression analyses to predict failing developmental milestones.

	Model 1			Model 2		
	B	SE B	OR (95% CI)	B	SE B	OR (95% CI)
Months past lower bound of age category	−0.16	0.12	0.86 (0.67–1.09)	−0.17	0.12	0.85 (0.67–1.07)
Mother’s education level	−0.35	0.29	0.70 (0.40–1.23)	−0.31	0.29	0.73 (0.42–1.29)
Without electricity	−0.86	0.66	0.42 (0.12–1.55)	−0.78	0.65	0.46 (0.13–1.65)
WHO height-for-age Z-score < −2	0.99	0.66	2.68 (0.73–9.79)			
Blood lead				0.20	0.11	1.23 (0.99–1.53)
